# Supplementary material for: Adrenergic modulation of melanocortin pathway by hunger signals
Source: Nat Commun. 2023 Oct 19;14:6602. doi: 10.1038/s41467-023-42362-8 (PMC10587058; doi:10.1038/s41467-023-42362-8)
Supplement: Supplementary file 1 — Supplementary Information [file 41467_2023_42362_MOESM1_ESM.pdf]

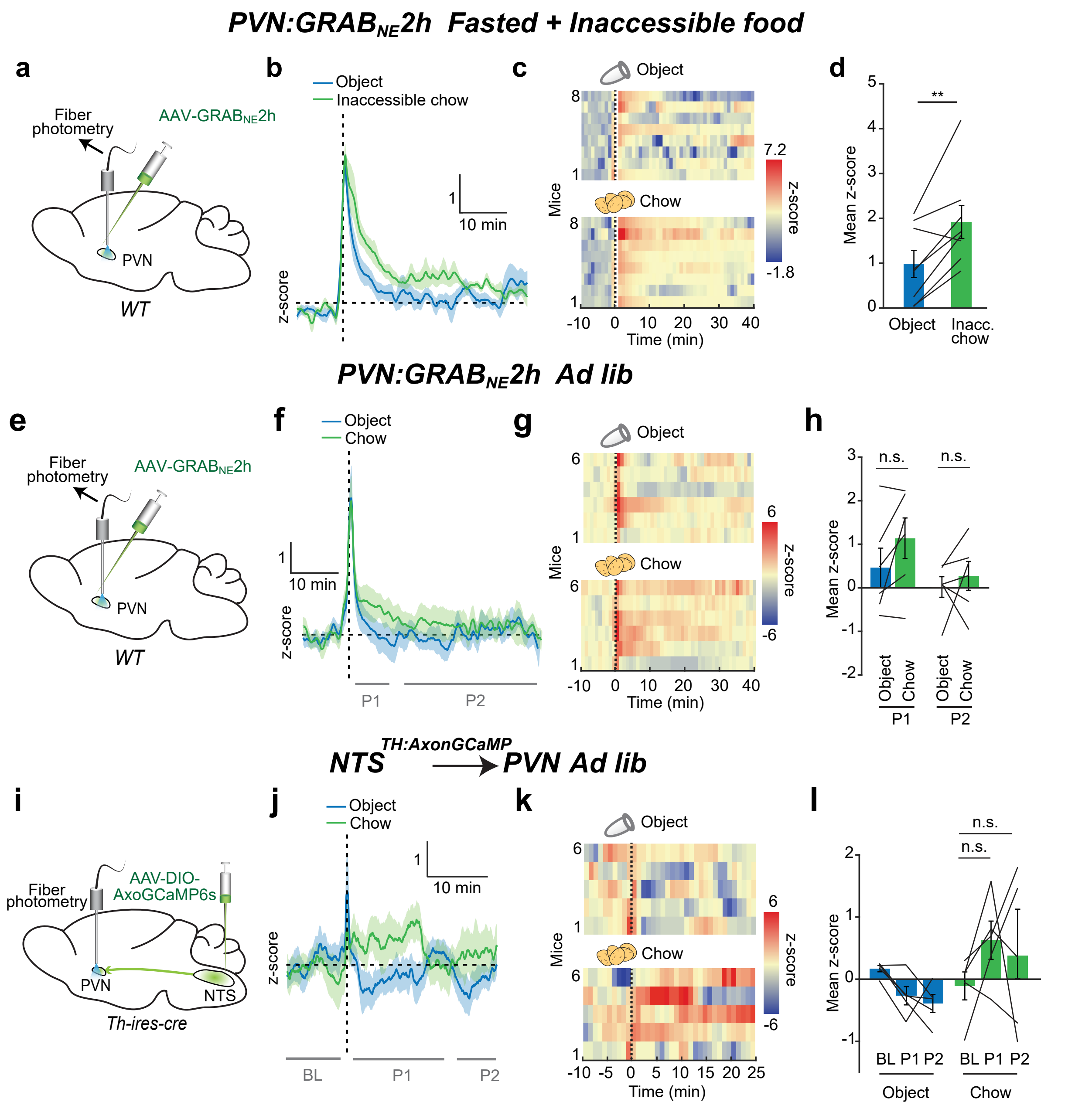

Supplementary Figure 1. The initial food-induced activation phase in PVN NE/E levels is sensory mediated and the food responsiveness is abolished in free feeding mice.

a Schematic of viral injection and fiber photometry-based NE/E measurement from PVN. **b,c** Average fiber photometry trace (b) and individual heat maps (c) showing $GRAB_{NE}2h$ signal in response to inaccessible chow food or inedible object. **d** Quantification of mean $GRAB_{NE}2h$ fluorescence in (b), n=8 mice, two-tailed, paired t-test, **p=0.0063. **e** Schematic of $GRAB_{NE}2h$ measurement from PVN. **f,g** Average line graph (f) and heat map of individual mouse (g) depicting change in $GRAB_{NE}2h$ signal over time in response to chow food or inedible object presentation. **h** Average bar graph of change in $GRAB_{NE}2h$ signal compared in response to object and chow presentation (n=6 mice, two-tailed paired t-tests, n.s.: p>0.08). **i-l** Same as in (e-h) except that NTS^{TH}→PVN projection activity is measured using axon-GCaMP expression in NTS^{TH} neurons, (n=5 mice, two-way RM-ANOVA with Tukey's multiple corrections, n.s.: p>0.58. Data are presented as mean values +/- SEM.

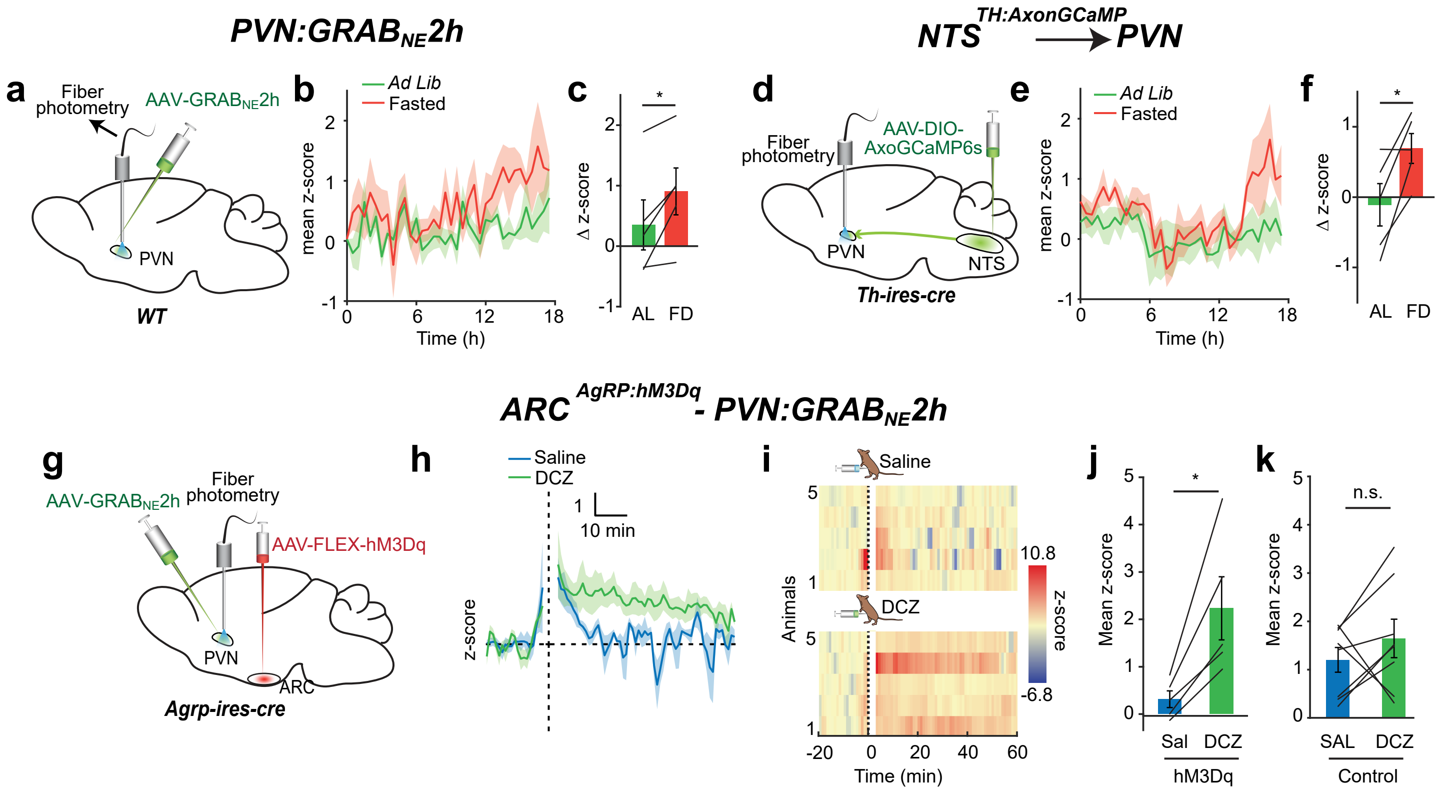

Supplementary Figure 2. Prolonged fasting elevates nor/adrenergic input to PVN.

a Schematic of GRAB$_{NE}$2h measurement from PVN. **b,c** Average line graph of chronic changes in NE/E signal in the presence and absence of food (n=5 mice, *p=0.034, two-tailed paired t-test). **d-f** Same as in a-c except that NTSTH→PVN projection activity is measured using axon-GCaMP expression in NTSTH neurons (n=5 mice, *p=0.027, two-tailed paired t-test). **g** Schematic of viral injections and fiber photometry-based NE/E measurement from PVN during chemogenetic AgRP stimulation. **h,i** Average fiber photometry trace (h) and individual heat maps (i) showing GRAB$_{NE}$2h response with and without concurrent AgRP neuron stimulation. **j,k** Quantification of GRAB$_{NE}$2h fluorescence in AgRP:hM3D expressing (j) and control (k, no DREADD) mice after saline or DCZ ip injection. (n=5 mice/group, *p=0.019 (AgRP:hM3D) and n.s.: p=0.37 (control), two-tailed paired t-test). Data are presented as mean values +/- SEM.

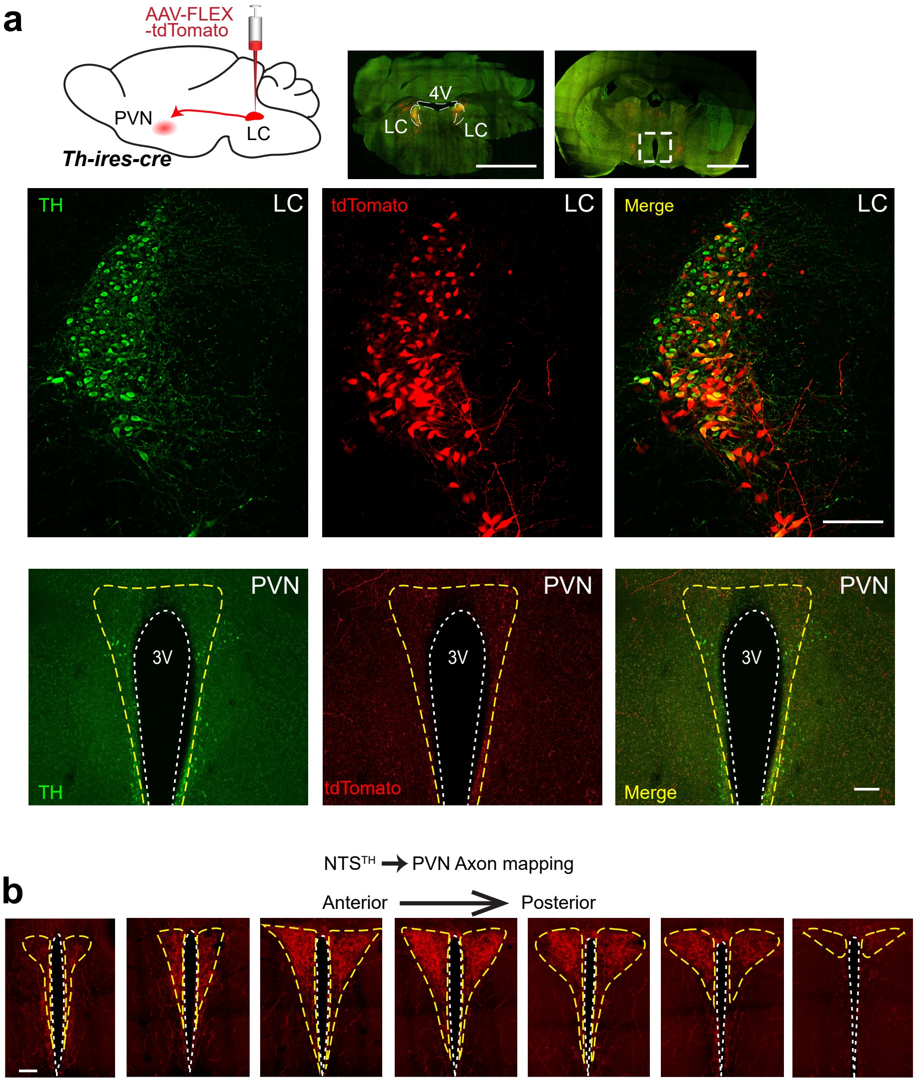

Supplementary Figure 3. PVN projection profiles of catecholaminergic hindbrain neurons.

a Schematic for assessing LC^{TH} projections using anterograde labeling with cre-dependent tdTomato. Representative images showing tdTomato (red) expression in tyrosine hydroxylase (green) positive LC neurons. Representative photomicrographs showing sparse PVN innervation by LC^{TH} neurons (scale bars: 2 mm, 100 μm and 200 μm). **b** Image showing robust NTS^{TH} projections (red) distributed throughout the rosto-caudal PVN (scale bar: 200 μm).

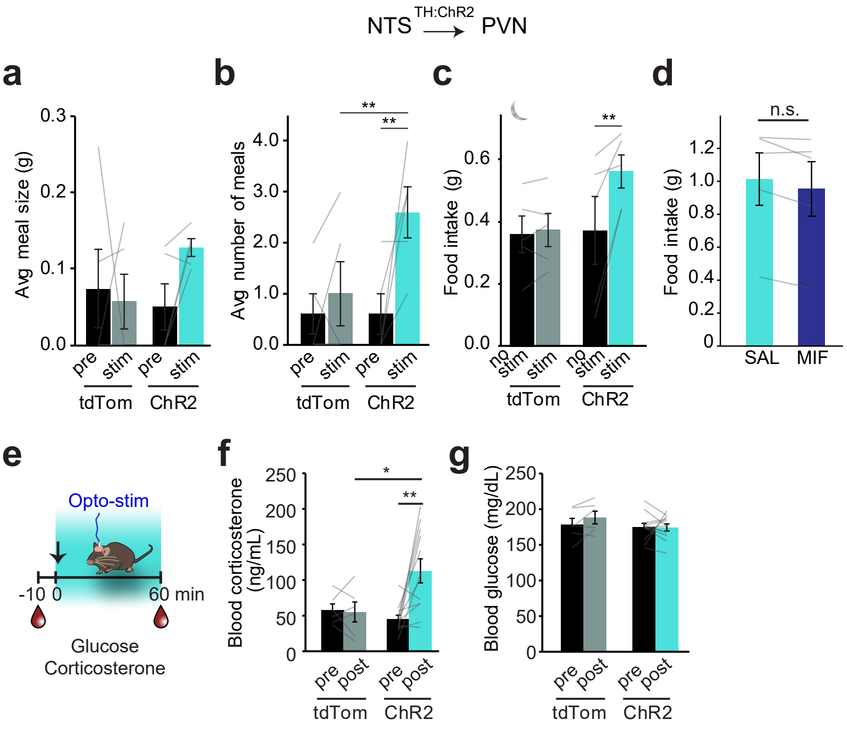

Supplementary Figure 4. NTSTH→PVN activation evokes feeding and corticosterone release.

a,b Effect of NTSTH→PVN optogenetic activation during light cycle on meal size (a) and number of meals (b) in control tdTomato (n=5) and ChR2 (n=5) expressing mice (2-way RM-ANOVA with Sidak's multiple comparison, tdTom stim vs ChR2 stim **p=0.0055, ChR2 stim vs ChR2 no stim **p=0.0024). **c** Effect of NTSTH→PVN activation on food intake at dark onset in control tdTomato (n=5 mice) and ChR2 (n=5 mice) animals (2-way RM ANOVA with Sidak's multiple comparison, **p=0.0071). **d** Effect of Mifepristone (5mg/kg) during NTSTH→PVN photostimulation on feeding response (two-tailed paired t-test, n.s.: p=0.069) **e** Experimental schematic for assessing effects of NTSTH→PVN photoactivation on circulating corticosterone and glucose levels. **f,g** Effect of 1h NTSTH→PVN stimulation on systemic (f) corticosterone and (g) glucose levels in control tdTomato (n=6) and ChR2 (n=12) transduced mice (2-way RM ANOVA with Sidak's multiple comparison, *p=0.0126, **p=0.0013). Data are presented as mean values +/- SEM.

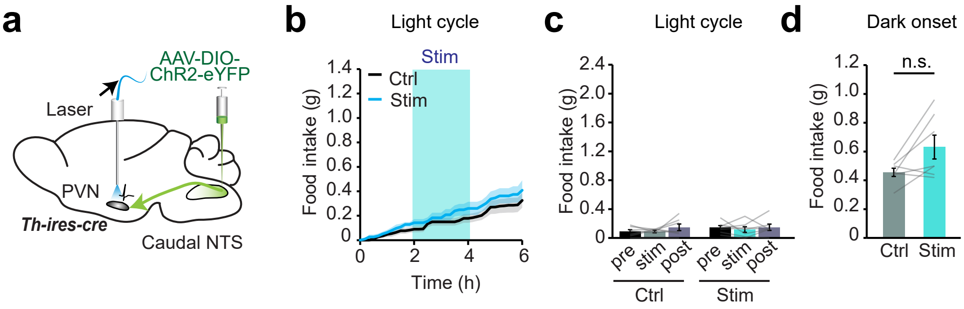

Supplementary Figure 5. Photostimulating caudal NTSTH →PVN projection does not affect food intake.

a Schematic depiction for activation of caudal NTSTH →PVN projection. **b,c** Cumulative food intake (b, n=7, two-tailed paired t-test) and average total food intake before, during and after photostimulation of cNTSTH →PVN projection (2 hours each) in free feeding mice in light phase (n=7, two-way RM-ANOVA). **d** Summary bar graph of 2-hours dark onset food intake with and without concurrent caudal cNTSTH →PVN projection stimulation (n=7 mice, two-tailed paired t-test, n.s.: p=0.057). Data are presented as mean values +/- SEM.

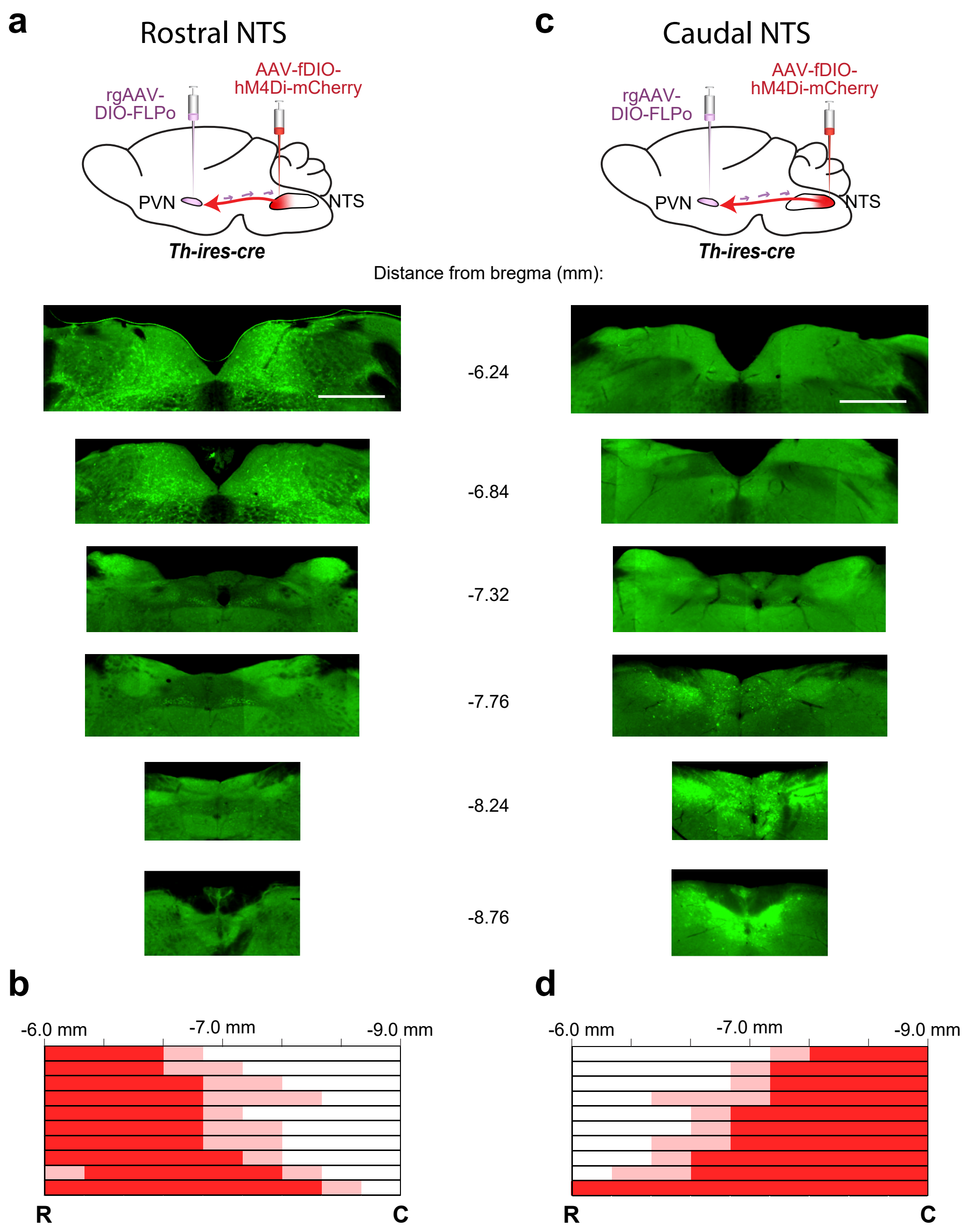

Supplementary Figure 6. Distribution of hM4Di expression in rostral and caudal NTSTH neurons.

a Schematic and representative images of hM4Di targeted to rostral NTSTH neurons. **b** Distribution of rostral NTS targeted hM4Di expression throughout the rostral-caudal axis of the NTS (mCherry signal was amplified with immunolabeling using a 488 secondary Ab). **c** Schematic for hM4Di injections into the caudal NTS (scale bars: 500 µm). **d** Distribution profile of hM4Di targeted to caudal NTS. Relative expression is indicated by color: dark red – high expression, light red – low expression, white – no expression.

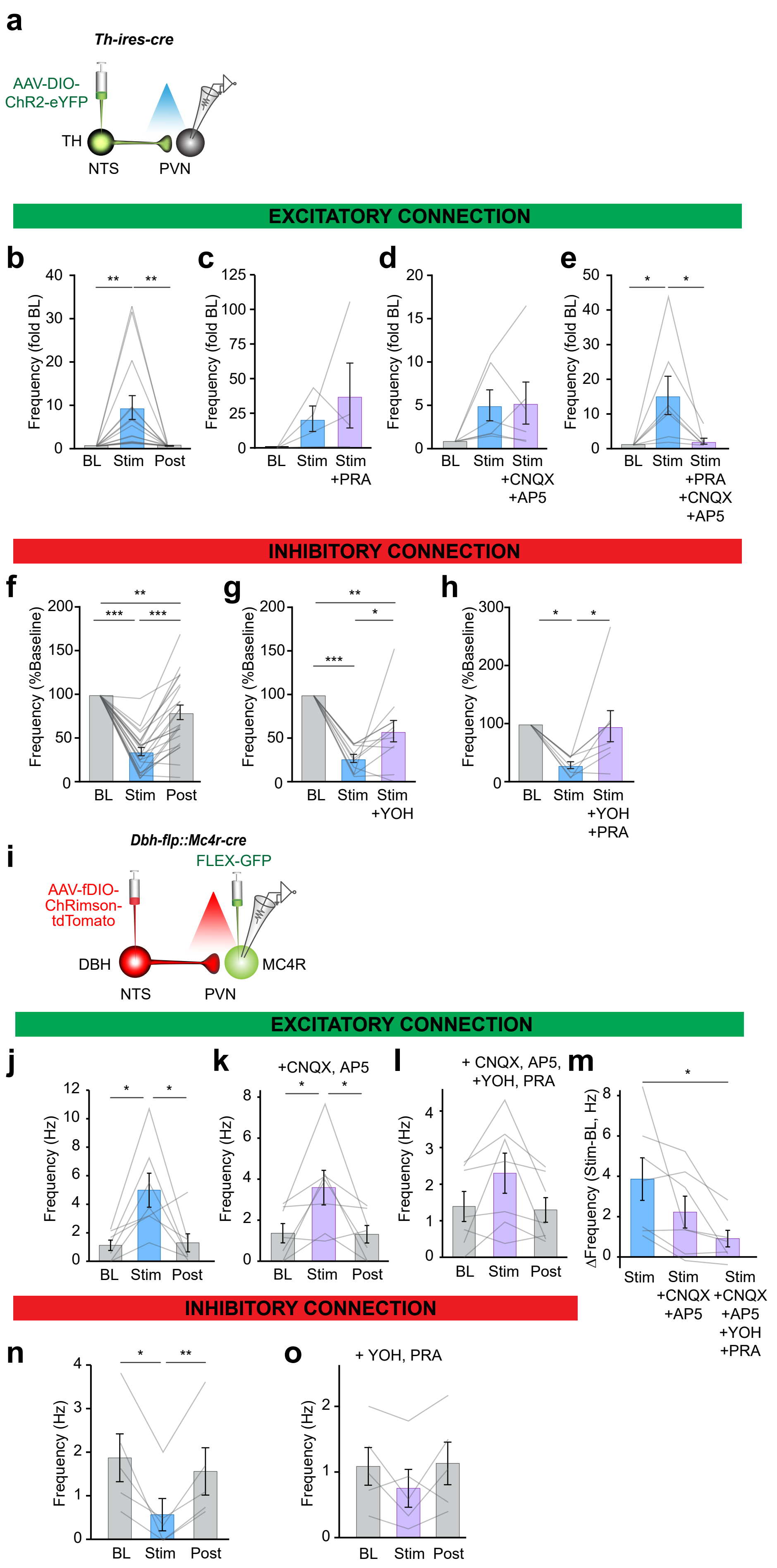
a
Th-ires-cre
AAV-DIO-ChR2-eYFP
TH
NTS
PVN
EXCITATORY CONNECTION
b
c
d
e
Frequency (fold BL)
BL
Stim
Post
+PRA
+CNQX
+AP5
INHIBITORY CONNECTION
f
g
h
Frequency (%Baseline)
+YOH
i
Dbh-flp::Mc4r-cre
AAV-fDIO-ChRimson-tdTomato
FLEX-GFP
DBH
MC4R
j
k
l
m
+CNQX, AP5
+ CNQX, AP5, +YOH, PRA
Frequency (Hz)
ΔFrequency (Stim-BL, Hz)
n
o
+ YOH, PRA

Supplementary Figure 7. NTSTH→PVN connection rely on nor/adrenergic signaling.

a Schematic for evaluating functional NTSTH→PVN connectivity using ChR2-assisted stimulation combined with loose seal recordings. **b-e** NTSTH axon stimulation excited a subset of PVN neurons (Two-way RM-ANOVA with Tukey's multiple comparisons). Effect of NTSTH photostimulation on PVN firing in control conditions (b, n=14 neurons, **p<0.0023), in the presence of prazosin (c, n=4 neurons), CNQX and AP5 (d, n=6 neurons), and prazosin, CNQX, and AP5 (e, n=7 neurons, *p<0.031). **f-h** Subset of PVN neurons inhibited by NTSTH projections (Two-way RM-ANOVA with Tukey's multiple comparisons). Effects of NTSTH→PVN inhibition in control conditions (f, n=22 neurons, **p=0.0087, ***p<0.0001), with bath application of yohimbine (g, n=11 neurons, *p=0.025, **p=0.0021, ***p<0.0001) and yohimbine and prazosin (h, n=8 neurons, *p<0.0192). **i** Schematic for evaluating functional NTSTH→PVN^{MC4R} connectivity using chrimson-assisted stimulation combined with loose seal recordings. **j-l** Subset of PVN^{MC4R} neurons activated by NTS^{DBH} axonal photostimulation (two-tailed paired t-tests). Response profile under control (j, n=7 neurons, *p<0.039), glutamatergic blockers CNQX and AP5 (k, n=7 neurons *p<0.040), and combination of glutamatergic and adrenergic blockers (l). **m** Summary of firing rate change as blockers added progressively (n=7 neurons, *p=0.027). **n,o** PVN^{MC4R} neurons inhibited by NTS^{DBH} axonal photostimulation (two-tailed paired t-tests). Response profile under conditions of control (n, n=5 neurons, *p=0.015, **p=0.0084) and α-adrenergic blockers yohimbine and prazosin (o, n=5 neurons, n=2-4 mice/group). Data are presented as mean values +/- SEM. For specific p-values, data and statistics, please see associated Source Data file.

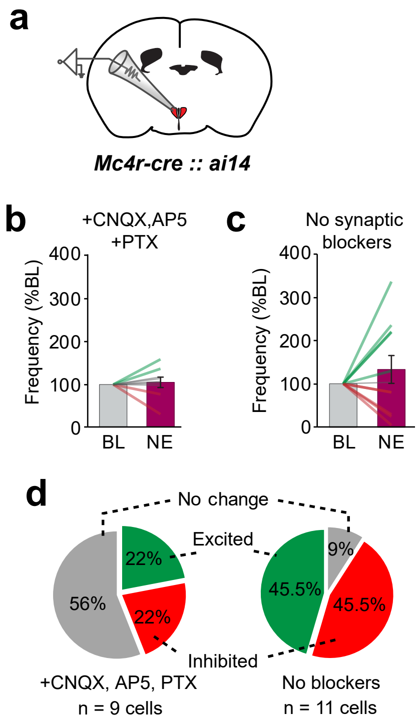

Supplementary Figure 8. Norepinephrine can directly and indirectly modulate PVN^{MC4R} neuron activity.

a Schematic for loose seal patch clamp from tdTomato labelled PVN^{MC4R} neurons. **b,c** Effect of NE on PVN^{MC4R} firing with (b, n=9 neurons, 3 mice) and without (c, n=10 neurons, 4 mice) synaptic blockade. **d** Pie chart summarizing effect of NE on PVN^{MC4R} neuron activity with and without synaptic blockade. Data are presented as mean values +/- SEM.

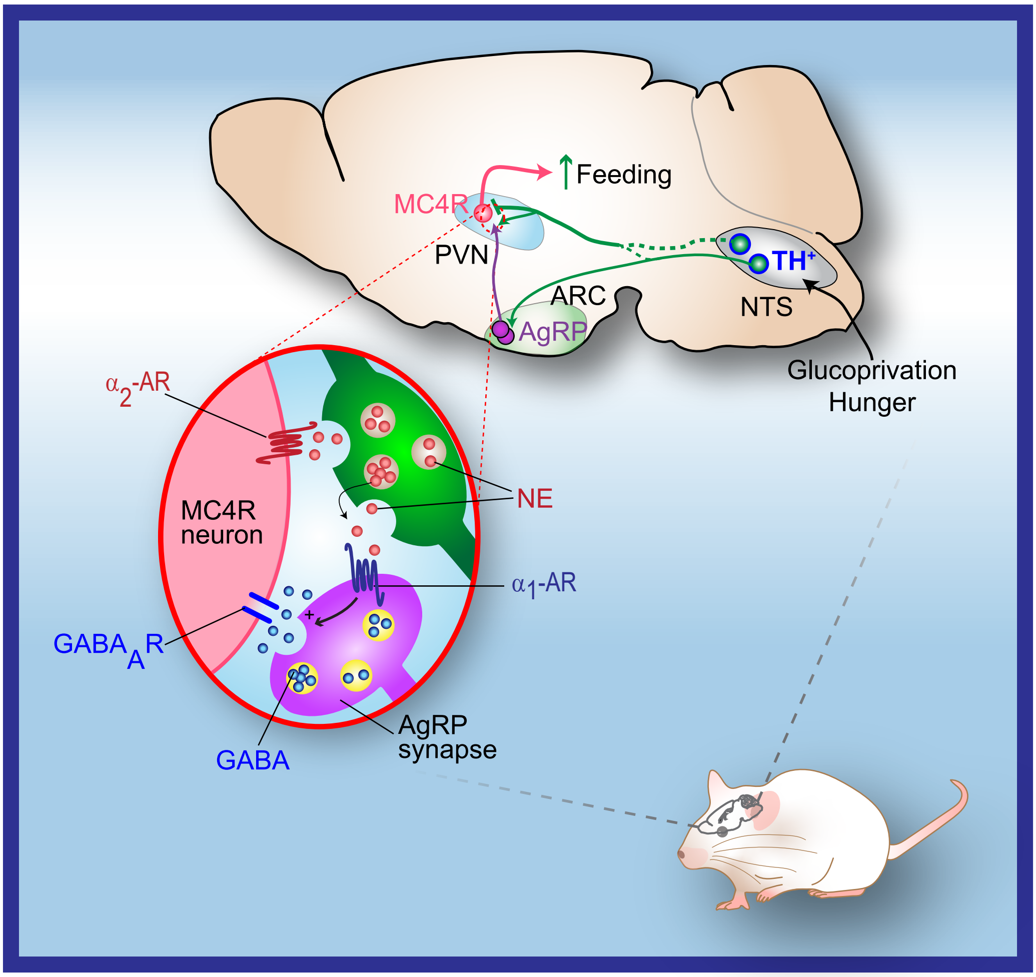

Supplementary Figure 9. A schematic model describing possible mechanism for nor/adrenergic modulation of melanocortin pathway.
