# Supplementary material for: Adrenergic modulation of melanocortin pathway by hunger signals
Source: Nat Commun. 2023 Oct 19;14:6602. doi: 10.1038/s41467-023-42362-8 (PMC10587058; doi:10.1038/s41467-023-42362-8)
Supplement: Supplementary file 3 — Reporting Summary [file 41467_2023_42362_MOESM3_ESM.pdf]

nature portfolio

Corresponding author(s): Deniz Atasoy

Last updated by author(s): 2023/09/28

Reporting Summary

Nature Portfolio wishes to improve the reproducibility of the work that we publish. This form provides structure for consistency and transparency in reporting. For further information on Nature Portfolio policies, see our Editorial Policies and the Editorial Policy Checklist.

Statistics

For all statistical analyses, confirm that the following items are present in the figure legend, table legend, main text, or Methods section.

n/a	Confirmed	
☐	☒	The exact sample size (*n*) for each experimental group/condition, given as a discrete number and unit of measurement
☐	☒	A statement on whether measurements were taken from distinct samples or whether the same sample was measured repeatedly
☐	☒	The statistical test(s) used AND whether they are one- or two-sided<br>*Only common tests should be described solely by name; describe more complex techniques in the Methods section.*
☐	☒	A description of all covariates tested
☐	☒	A description of any assumptions or corrections, such as tests of normality and adjustment for multiple comparisons
☐	☒	A full description of the statistical parameters including central tendency (e.g. means) or other basic estimates (e.g. regression coefficient) AND variation (e.g. standard deviation) or associated estimates of uncertainty (e.g. confidence intervals)
☐	☒	For null hypothesis testing, the test statistic (e.g. *F*, *t*, *r*) with confidence intervals, effect sizes, degrees of freedom and *P* value noted<br>*Give P values as exact values whenever suitable.*
☒	☐	For Bayesian analysis, information on the choice of priors and Markov chain Monte Carlo settings
☒	☐	For hierarchical and complex designs, identification of the appropriate level for tests and full reporting of outcomes
☐	☒	Estimates of effect sizes (e.g. Cohen's *d*, Pearson's *r*), indicating how they were calculated

Our web collection on statistics for biologists contains articles on many of the points above.

Software and code

Policy information about availability of computer code

Data collection	Slide scanner images were acquired using VS2000 (Olympus).<br>Fiber photometry data were acquired using Doric Neuroscience Studio v5.4.1.23.<br>Food intake data was acquired using Coulbourn Habitest system or FED3 devices.
Data analysis	lectrophysiology data were analyzed using Molecular Devices Clampfit (10.2 & 10.6).<br>Images were analyzed using Olympus OlyVIA v3.2.1.<br>Custom MATLAB (R2020b) script was used to analyze the fiber photometry data acquired by Doric Lenses BFP fiber photometry systems, and to analyze the feeding data acquired with FED3 (Open Ephys) devices. The scripts are available upon request.<br>Statistical tests were calculated using GraphPad Prism 9.<br>Behavior data was analyzed using Ethovision XT15 software.

For manuscripts utilizing custom algorithms or software that are central to the research but not yet described in published literature, software must be made available to editors and reviewers. We strongly encourage code deposition in a community repository (e.g. GitHub). See the Nature Portfolio guidelines for submitting code & software for further information.

Data

Policy information about availability of data

All manuscripts must include a data availability statement. This statement should provide the following information, where applicable:

- Accession codes, unique identifiers, or web links for publicly available datasets
- A description of any restrictions on data availability
- For clinical datasets or third party data, please ensure that the statement adheres to our policy

The data generated during and/or analyzed during the current study are available in the Source Data file spreadsheets associated with each figure.

Research involving human participants, their data, or biological material

Policy information about studies with human participants or human data. See also policy information about sex, gender (identity/presentation), and sexual orientation and race, ethnicity and racism.

Reporting on sex and gender	N/A
Reporting on race, ethnicity, or other socially relevant groupings	N/A
Population characteristics	N/A
Recruitment	N/A
Ethics oversight	N/A

Note that full information on the approval of the study protocol must also be provided in the manuscript.

Field-specific reporting

Please select the one below that is the best fit for your research. If you are not sure, read the appropriate sections before making your selection.

☒ Life sciences ☐ Behavioural & social sciences ☐ Ecological, evolutionary & environmental sciences

For a reference copy of the document with all sections, see nature.com/documents/nr-reporting-summary-flat.pdf

Life sciences study design

All studies must disclose on these points even when the disclosure is negative.

Sample size	Sample size (number of animals) was determined depending on the data type and by the standards accepted in the field. Sample size was determined based on experience from previous studies and we did not perform power analysis.
Data exclusions	Data from animals where the virus injection or fiber tip was off target were removed from the study. Also, data from animals whose FED3 feeder device jammed or whose fibers broke during data acquisition were removed from that specific analysis.<br>All Data exclusion criteria were pre-determined.
Replication	Group sample sizes were provided in the respective figure legends. On average, 4-8 animals were used for data analysis.<br>Experiments were repeated in independent separate cohorts and combined for final analysis.
Randomization	Mice were randomly allocated into each experimental group.
Blinding	Blinding was not possible during initial planning of animal selection to ensure the groups of animals were age and sex matched. When applicable, the investigators were blinded for the experiments and data analyses.

Reporting for specific materials, systems and methods

We require information from authors about some types of materials, experimental systems and methods used in many studies. Here, indicate whether each material, system or method listed is relevant to your study. If you are not sure if a list item applies to your research, read the appropriate section before selecting a response.

Materials & experimental systems

n/a	Involved in the study
☐	☒ Antibodies
☒	☐ Eukaryotic cell lines
☒	☐ Palaeontology and archaeology
☐	☒ Animals and other organisms
☒	☐ Clinical data
☒	☐ Dual use research of concern
☒	☐ Plants

Methods

n/a	Involved in the study
☒	☐ ChIP-seq
☒	☐ Flow cytometry
☒	☐ MRI-based neuroimaging

Antibodies

Antibodies used

Primary antibodies : Rabbit anti-TH (1:5000, Abcam, #ab112); Rabbit anti-cFos (1:5000, Cell signaling, #2250); Rabbit anti-mCherry (1:1000, Abcam, #ab167453).
Secondary antibodies: Goat anti-rabbit IgG (H+L) Alexa Flour 488 / 568 (1:500, Thermo-Fisher)

Validation

Commercial antibodies were validated by the manufacturer.

Animals and other research organisms

Policy information about studies involving animals; ARRIVE guidelines recommended for reporting animal research, and Sex and Gender in Research

Laboratory animals

6-24 weeks old male and female mice were used for each experiment and strain. Mice were kept at room temperature (20-24 C) at 40-60% humidity.
Mice used in this study:
Sim1-cre (Jackson Labs Stock 006395),
Th-cre (Jackson Labs Stock 008601),
Th-ires-cre (Lindeberg et al., 2004),
Agrp-ires-cre (Jackson Labs Stock 012899),
Ai14 (Jackson Labs Stock 007914),
Mc4r-cre (Jackson Labs Stock 030759),
Dbh-flp (Jackson Labs Stock 033952),
C57BL/6 (Jackson Labs Stock 000664)

Wild animals

Wild animals were not used in this study.

Reporting on sex

For each experiment, similar sizes of male and female mice were used and aggregated in the study. Based on literature and our previous studies, there was no reason to expect sexually dimorphic phenotypes, therefore we did not perform sex-specific analyses.

Field-collected samples

Field-collected samples were not used in this study.

Ethics oversight

Animal care and experimental procedures were approved by University of Iowa Institutional Animal Care and Use Committee (IACUC, Protocol number: 1082183), and Istanbul Medipol University MEDITAM. Mice welfare and health checks were conducted in accordance with the (IACUC) guidelines.

Note that full information on the approval of the study protocol must also be provided in the manuscript.
